# 92 Enhancing Self-Efficacy for and Adherence to Resistance Training in Older Adults: A Multi-Stakeholder Perspective of a Supervised-to-Unsupervised Step-Down Model

**DOI:** 10.1093/eurpub/ckae114.061

**Published:** 2024-09-26

**Authors:** Brian Mulhare, Ciara O’Hagan, Michael Harrison, Sarah Fagan, Bróna Kehoe

**Affiliations:** South East Technological University, Waterford, Ireland; South East Technological University, Carlow, Ireland; South East Technological University, Waterford, Ireland; South East Technological University, Waterford, Ireland; South East Technological University, Waterford, Ireland

## Abstract

**Background:**

With the ageing population, it is increasingly important to support older adults in maintaining good health and independence. Resistance exercise has been proven to have physical, physiological, and cognitive benefits. However, only a small percentage of older adults participate in sufficient muscle-strengthening activities. Community or home-based programs may offer the best mode to address this, but lack long-term sustainability. A model combining both approaches could reinforce meaningful behavioural change. Expert consultation is necessary to identify potential challenges in this approach.

**Methods:**

A multi-stakeholder expert panel (n = 11) of academics, facilitators, and older adults was consulted to critically evaluate a step-down exercise model using a multi-stage framework. Initially, the panel was briefed with an executive summary of a behavioural analysis of resistance training in older adults based on formative qualitative research (n = 27). In the first stage, the panel engaged in a structured process to outline the intervention’s key components, identify potential difficulties, and develop solutions through voting. The second stage focused on determining necessary content and resources, employing a silent generation technique for devising solutions, followed by group discussions and round-robin sharing to address implementation challenges. This was audio-recorded, transcribed and thematically analysed to identify the barriers and facilitators to the implementation of a step-down model.

**Results:**

The primary themes were: Messaging, Health Autonomy, Education, Tiers/Levels, Individual Exerciser, and Equity and Scalability. Challenges include poor messaging to older adults about resistance training, leading to a lack of awareness, misconceptions and poor engagement. The approach to healthcare and social welfare fosters dependency, hindering self-responsibility and health literacy. Starting with a supervised group setting supports education and behavioural change, and allows intensive guidance with unfamiliar exercise to build self-efficacy, eventually transitioning to more independent, remotely-supported exercise. The tiered approach was perceived to gradually reduce dependency whilst providing benchmarks for progress. However, there were concerns about the suitability for unsupervised high-intensity exercise and acceptance of independent exercise among older adults.

**Conclusion:**

Multi-stakeholder perspectives in the development of complex interventions are valuable to exploring potential implementation challenges and solutions, thereby enhancing the feasibility, acceptance, and, hence, potential success of the intervention.

